# Reinforcing the role of magnesium in preventing cisplatin-induced nephrotoxicity: real-world evidence and clinical implications

**DOI:** 10.1093/ckj/sfaf212

**Published:** 2025-07-07

**Authors:** Luca Nardelli, Arjunmohan Mohan, Sandra M Herrmann

**Affiliations:** Division of Nephrology, Dialysis and Kidney Transplantation, Fondazione IRCCS Ca’ Granda Ospedale Maggiore Policlinico, Milan, Italy; Department of Clinical Sciences and Community Health, Università degli studi di Milano, Milan, Italy; Department of Internal Medicine, Division of Nephrology and Hypertension, Mayo Clinic, Rochester, MN, USA; Department of Internal Medicine, Division of Nephrology and Hypertension, Mayo Clinic, Rochester, MN, USA

Cisplatin remains a mainstay in cancer treatment, but its nephrotoxicity often forces dose reductions, treatment delays or discontinuation. Cisplatin-associated acute kidney injury (CP-AKI) represents a threatening complication that increases morbidity and mortality. While limited strategies exist to prevent CP-AKI, growing evidence supports the protective role of magnesium supplementation [1–4].

In a recent multicenter retrospective cohort study, Gupta *et al*. examined whether prophylactic intravenous (IV) magnesium could reduce the incidence of CP-AKI or death [5]. The study included over 13 700 adult patients, not on kidney replacement therapy (KRT), who received their first dose of IV cisplatin between 2006 and 2022. Of these, 28.4% received IV magnesium (median dose 2 g) on the day of cisplatin administration.

The primary outcome—a composite of AKI [defined as a ≥2-fold increase in serum creatinine (SCr) or need for KRT] or death within 2 weeks—was significantly lower in the magnesium group. IV magnesium was associated with 20% lower odds of CP-AKI or death (odds ratio 0.80; 95% confidence interval 0.66–0.97), with stronger protective effects observed in patients aged <65 years, women, diabetics, those with baseline eGFR ≥90 mL/min/1.73 m² and higher baseline magnesium levels (2.0–2.2 mg/dL) [5].

**Figure 1:**
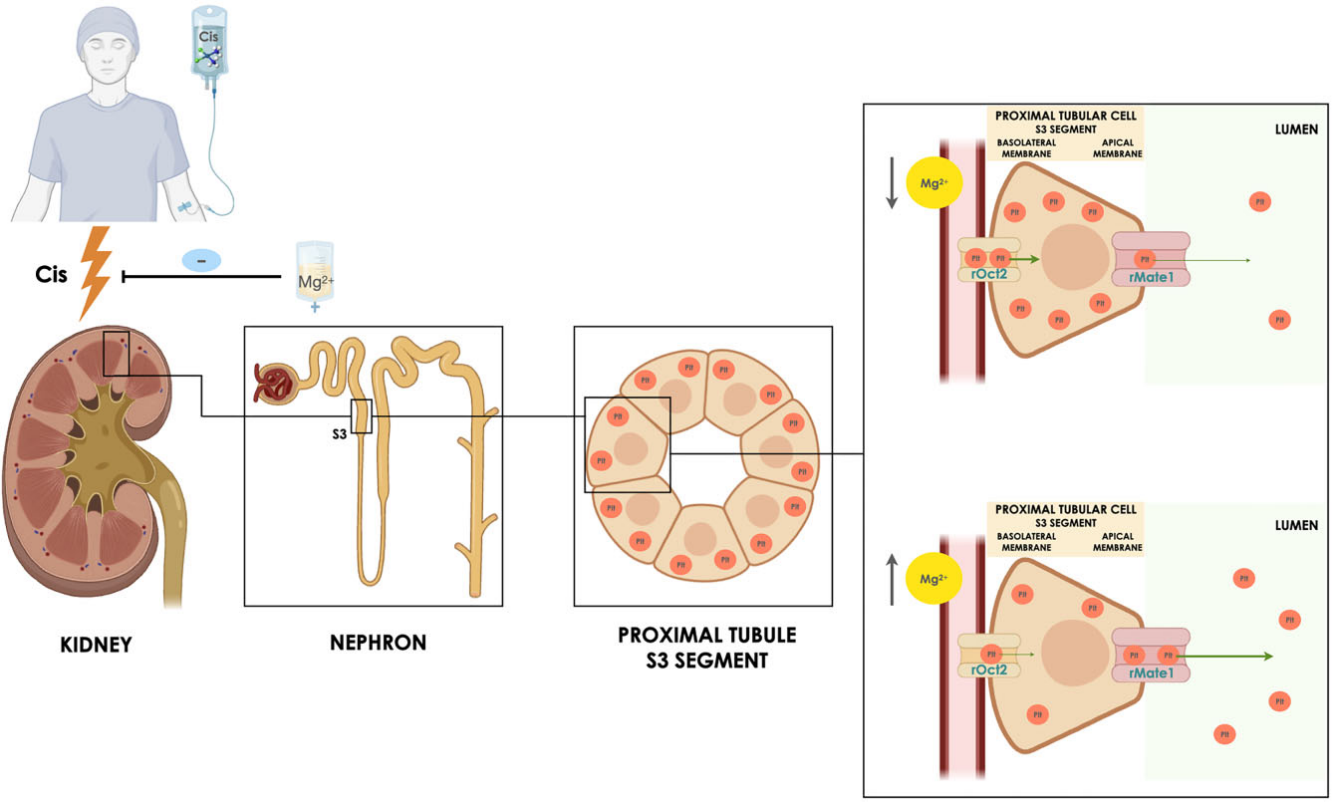
Mechanisms of cisplatin-induced nephrotoxicity and nephroprotection mediated by magnesium premedication. Platinum renal pharmacokinetics is mainly modulated by rOct2 and rMate1. rOct2, which is predominantly localized in the basolateral membranes of proximal tubules, is expressed magnesium-dependently and hypomagnesemia causes upregulation of rOct2. Conversely, rMate1, which is present in the apical membranes, transports platinum from the proximal tubule into the urine, and hypomagnesemia induces its downregulation. These intracellular mechanisms eventually result in intracellular platinum accumulation and tubular toxicity. Cis, cisplatin; Mg^2+^, magnesemia; rOct2, organic cation transporter 2; Plt, platinum; rMAte1, multidrug and toxin extrusion protein 1.

Mechanistically, magnesium may limit platinum accumulation in kidney tubular cells, likely by modulating renal transporters involved in drug uptake and clearance (Fig. 1) [1–4]. Gupta *et al*. noted a possible threshold effect, with greater protection in patients with higher baseline serum magnesium. This raises the question of whether current dosing (typically 1–2 g) is adequate and whether weight-based or tailored dosing might offer enhanced protection, especially for those with lower baseline magnesium [6].

However, the primary outcome was largely based on changes in SCr, raising concerns that patients with high baseline eGFR (≥90 mL/min/1.73 m²) may experience a significant kidney function decline without a corresponding rise in SCr, due to their greater renal reserve. Furthermore, the study did not explicitly assess outcomes across stages of chronic kidney disease (CKD), though magnesium handling and cisplatin pharmacokinetics might differ in these populations. Longer term effects—such as CKD progression after CP-AKI—were not assessed and warrant further study [5].

**Figure 2:**
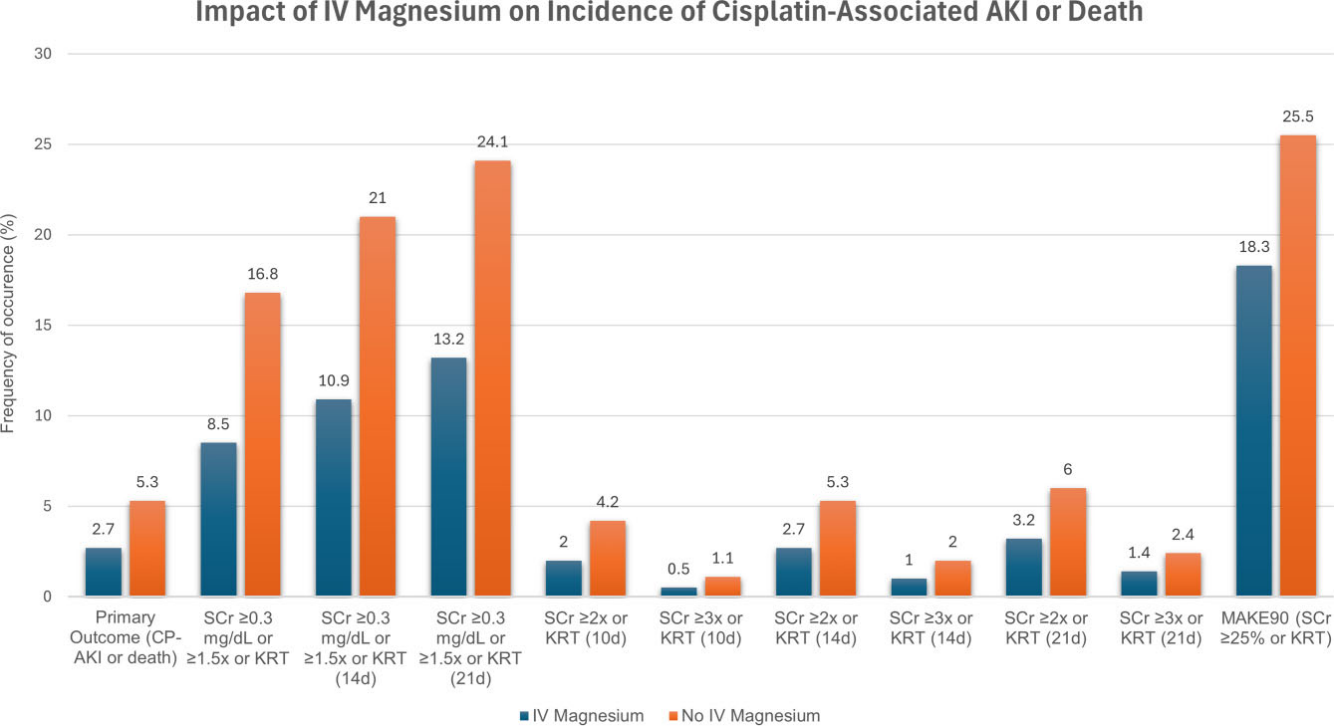
Impact of IV magnesium on incidence of CP-AKI or death. In the multicenter retrospective cohort study by Gupta *et al*. [5] the benefit of IV magnesium was consistent across several sensitivity and secondary analyses, including stricter and more liberal definitions of AKI.

Importantly, IV magnesium was well tolerated, with no increase in adverse events and its benefit was consistent across several sensitivity and secondary analyses (Fig. 2). In clinical practice, IV magnesium is not uniformly given with cisplatin. Yet, these findings may shift practice, particularly for patients with low baseline magnesium. Some clinicians already feel more inclined to administer magnesium prophylactically in such cases, though adoption is not uniform—likely reflecting the limitations inherent in retrospective evidence.

Given its safety, affordability and mechanistic plausibility, these findings support considering IV magnesium as a routine prophylactic measure in cisplatin protocols. Although causality remains unproven, the study's large sample and consistent subgroup effects lend clinical weight. Prospective randomized trials are needed to clarify optimal dosing, long-term benefits and effectiveness in high-risk patients.
